# Can accuracy of component alignment be improved with Oxford UKA Microplasty® instrumentation?

**DOI:** 10.1186/s13018-020-01868-3

**Published:** 2020-08-26

**Authors:** Jonathan Patrick Ng, Jason Chi Ho Fan, Lawrence Chun Man Lau, Tycus Tao Sun Tse, Samuel Yik Cheung Wan, Yuk Wah Hung

**Affiliations:** Department of Orthopaedics and Traumatology, Alice Ho Miu Ling Nethersole Hospital, 11 Chuen On Road, Hong Kong, Hong Kong SAR

**Keywords:** Unicompartmental knee arthroplasty, Fixed bearing, Mobile bearing, Instrumentation, Component alignment

## Abstract

**Background:**

One factor in the long-term survivorship of unicompartmental knee arthroplasty is the accuracy of implantation. In addition to implant designs, the instrumentation has also evolved in the last three decades to improve the reproducibility of implant placement. There have been limited studies comparing mobile bearing unicompartmental knee arthroplasty with contemporary instrumentation and fixed bearing unicompartmental knee arthroplasty with conventional instrumentation. This study aims to determine whether the Microplasty instrumentation in Oxford unicompartmental knee arthroplasty allows the surgeon to implant the components more precisely and accurately.

**Methods:**

A total of 150 patients (194 knees) were included between April 2013 and June 2019. Coronal and sagittal alignment of the tibial and femoral components was measured on postoperative radiographs. Component axial rotational alignment was measured on postoperative computer tomography. The knee rotation angle was the difference between the femoral and tibial axial rotation. A rotational mismatch was defined as a knee rotation angle of > 10°. Statistical analysis was performed using Student *t* test and Mann-Whitney nonparametric test. A *p* value < 0.05 was considered statistically significant in each analysis.

**Results:**

Between April 2013 to June 2019, 112 patients (150 knees) received Oxford unicompartmental knee arthroplasty, one patient (2 knees) had Journey unicompartmental knee arthroplasty, and 37 patients (42 knees) received Zimmer unicompartmental knee arthroplasty. All femoral components in the Oxford group were implanted within the reference range, compared with 36.6% in the fixed bearing group (*p* < 0.001). 88.3% of Oxford knees had tibial component falling within the reference range, whereas 56.1% of knees in the fixed bearing group fell within the reference range (*p* < 0.001). 97.5% of Oxford knees had tibial slope that fell within reference range, whereas 53.7% fell within range for fixed bearing group (*p* < 0.001). Femorotibial rotational mismatch of more than 10° was noted in 13.8% in Oxford group and 20.5% in fixed bearing group (*p* = 0.04).

**Conclusion:**

In conclusion, Microplasty instrumentation for Oxford mobile bearing unicompartmental knee arthroplasty is more accurate and precise compared to conventional fixed bearing unicompartmental knee arthroplasty in sagittal, coronal, and axial alignment. Prospective studies with long-term follow-up are warranted to investigate the clinical implications.

## Introduction

Unicompartmental knee arthroplasty (UKA) is an established treatment option for patients with isolated medial or lateral compartment knee osteoarthritis. Studies with long-term follow-up have demonstrated fewer complications and faster recovery compared with total knee arthroplasty (TKA) [1–3]. One factor in the long-term survival of UKA is the accuracy of implantation, and inaccurate implantation rates of up to 30% have been reported [4–6].

Two fundamentally different design concepts exist for UKA, fixed bearing and mobile bearing, each with its advantages and disadvantages. Specific to mobile bearing design is the risk of bearing dislocation, a devastating complication that requires reoperation, and is associated with component malpositioning and improper ligament balancing [7–10]. In light of this, the Oxford mobile bearing UKA has made some changes to its instrumentation in an attempt to improve its accuracy and precision in implant positioning. In contrast to the separate femoral and tibial saw guides in the conventional instrumentation in fixed bearing UKA, the phase III Oxford UKA Microplasty® instrumentation features a linkage of the tibial saw guide, the G clamp and the femoral sizing spoon; which reflects the axis of the medial femoral condyle, proposedly allowing for more consistent and accurate tibial resection [11]. In addition, the femoral component is milled with a spherical cutter in the Oxford UKA, rather than a conventional cutting jig and oscillating saw. A final spherical cut preserves equal stresses to the polyethylene layer, even if rotated in the frontal plane [11]. Therefore, in addition to higher instrumentation precision, the Oxford UKA also has the theoretical advantage of adjusting for small amount of rotational mismatch, thus accepting a larger margin of error in implant positioning.

The Oxford UKA designer series reported a 10-year survivorship of 96% [12]. Similar results were reproduced in other independent centers, as reflected in a recent systematic review of 8658 knees, with a 10-year survival of 93% and 15-year survival of 89% [13]. Compared with conventional fixed bearing designs, a recent systematic and meta-analysis showed no differences between the two implant designs, with regard to survivorship and functional outcomes [14]. However, the majority of the studies included were limited to small sample sizes, with relatively short-term follow-up. More importantly, the majority of studies compared the results of older Oxford mobile bearing designs and instrumentation rather than the Microplasty® instrumentation. Therefore, the superiority of mobile bearing with new instrumentation versus fixed bearing UKA with conventional instrumentation remains a topic of debate. This study aims to determine whether the Microplasty® instrumentation in Oxford UKA allows the surgeon to implant the components more precisely and accurately.

## Methods

This was a retrospective review of prospectively collected data in a joint replacement center-based registry. Between April 2013 and June 2019, our center performed 194 primary medial UKAs in 112 patients. Of which, 112 patients (150 knees) received phase III mobile bearing cemented Oxford knee (Zimmer Biomet, Warsaw, Indiana) with Microplasty® instrumentation via a minimally invasive approach, one patients (2 knees) was implanted with Journey UKA (Smith and Nephew), and 37 patients (42 knees) received Zimmer Unicompartmental High Flex Knee System (Zimmer Inc., Warsaw, Indiana).

The indications for surgery were a confirmed diagnosis of isolated anteromedial osteoarthritis (AMOA), correctable varus deformity, a preoperative range of knee flexion greater than 100°, flexion contracture of less than 10°, and a clinically stable knee in the frontal and sagittal planes. Absolute contraindications for surgery included symptomatic patellofemoral disease or grooving of lateral patellofemoral joint, incompetent anterior cruciate ligament (ACL), or presence of inflammatory disease. The final judgment to proceed with UKA was intraoperative confirmation of an intact ACL and lateral compartment. The decision to use either a fixed or mobile bearing UKA was not randomized but rather based on the availability of the implants and equipment.

All patients received similar postoperative rehabilitation protocols and were evaluated at 1, 6, and 12 months of follow-up and then annually. Implant revision and reoperations were recorded. The patients were categorized into two groups for analysis: Oxford UKA and conventional fixed bearing UKA. Postoperative radiographs and computed tomography (CT) using a 64-row multislice CT system were performed. The radiographs closest to the postoperative 1-year timepoint were selected to assess alignment.

On anteroposterior (AP) views, the varus/valgus alignment of the femoral and tibial components were measured. On lateral views, the tibial slope was measured relative to anterior cortex of the tibia. Femoral and tibial axial rotation was obtained on CT axial cuts. Rotation of the femoral component was measured by using the transepicondylar axis (TEA) as reference. Reference for the tibial rotation was a line perpendicular to the posterior tibial cortex. The knee rotation angle (KRA) was the difference between the femoral and tibial axial rotation. A rotational mismatch was defined as a KRA of > 10° [15].

The radiographs were measured by two independent observers. The intra- and interobserver reliabilities of each measurement were assessed using an intraclass correlation coefficient (ICC). The intra- and interobserver reliability for all radiographic measurements were considered acceptable, ranging from 0.86 to 0.99 and 0.93 to 0.99, respectively. The reference ranges for each component alignment were determined according to the manufacturers’ recommendations. Statistical analysis using the statistical package SPSS for Windows (v.15.0, Chicago, IL, USA) was performed using Student *t* test and Mann-Whitney nonparametric test. A *p* value < 0.05 was considered statistically significant in each analysis.

## Results

Of the 194 medial UKAs performed between April 2013 and June 2019, 150 were mobile bearing and 44 were conventional fixed bearing. All the mobile bearings were performed with Oxford Microplasty® instrumentation and all fixed bearing were performed with conventional instrumentation. There was a significant difference for time to follow-up (*p* < 0.005) because the center implanted the fixed bearing and the mobile bearing consecutively over time (Table 1). Furthermore, there was also a significant difference in operation time (*p* = 0.03). Three patients (3 knees, 1.5%) who underwent Zimmer UKA were complicated by fractures of the medial tibial condyle requiring revision to TKA within 60 days of index operation. There was one culture-negative periprosthetic infection (0.7%) in the Oxford mobile bearing group which required reoperation for washout and exchange of insert.

**Table 1:** Baselines characteristics in the mobile bearing and fixed bearing groups

Parameter	Mobile bearing group (***n*** = 150)	Fixed bearing group (***n*** = 44)
Age (mean ± SD)	68.6 ± 6.9	67.7 ± 7.1
Gender (male to female)	71:79	6:38
Ahlbäck grade		
Grade 1	12	12
Grade 2	129	32
Grade 3	9	0
Operation time	80.2 min	106.4 min
Mean follow-up (months)	14.4 months	36.1 months

**Fig. 1:**
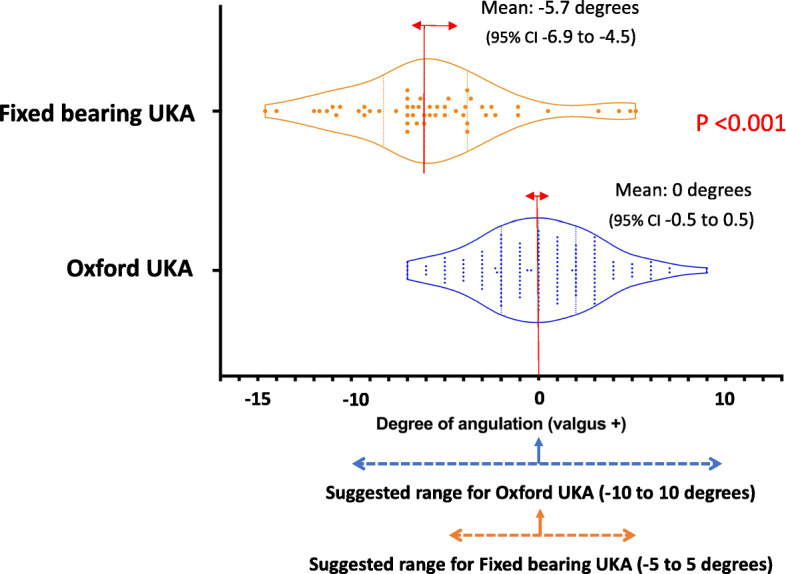
Comparison of femoral component coronal alignment

Postoperative CT was available in 80 patients (53.3%) with Oxford UKA, and 36 (94.7%) patients with fixed bearing UKA. The mean femoral component alignment for Oxford UKA was 0° (i.e. neutral to mechanical axis) (95% CI 0.5° valgus to 0.5° varus). All Oxford UKA were implanted within the reference range of 0 ± 10° as recommended by the manufacturer’s criteria. The mean femoral component alignment for conventional fixed bearing UKA (Zimmer UKA and Journey UKA) was 5.7° varus (95% CI 4.5 to 6.9° varus). In contrast to Oxford UKA, 26 fixed bearing UKA (63.4%) were implanted outside of the reference range of 0 ± 5° (*p* < 0.001) (Fig. 1).

**Fig. 2:**
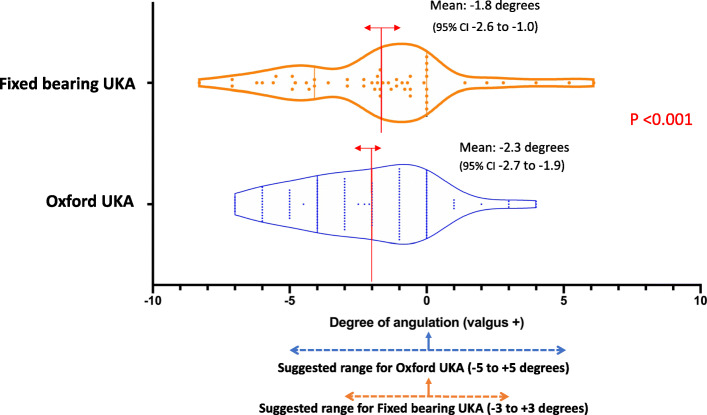
Comparison of tibial component coronal alignment

**Fig. 3:**
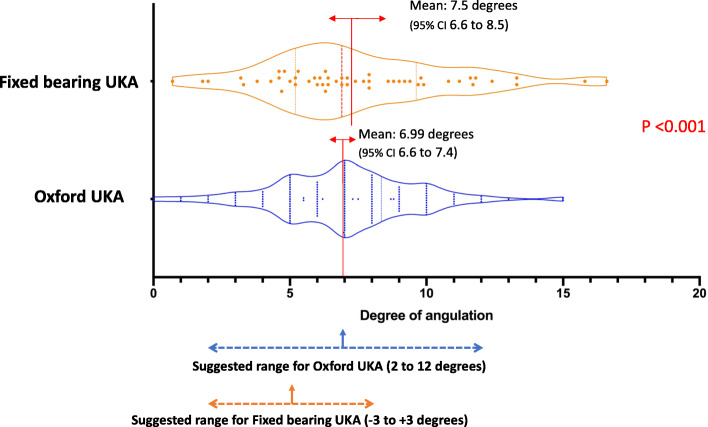
Comparison of posterior tibial slope

The mean tibial component alignment for Oxford UKA was 2.3° varus (95% CI 1.6 to 2.7° varus), with 88.3% falling within the reference range. The mean tibial alignment for fixed bearing UKA was 1.8° varus (95% CI 1 to 2.6° varus), with 23 knees (56.1%) falling within the reference range (*p* < 0.001) (Fig. 2). In the sagittal plane, the mean tibial slope was 7° for Oxford UKA (95% CI 6.6–7.4), with 117 (97.5%) of knees falling within the recommended range. The mean tibial slope for fixed bearing UKA was 7.5° (95% CI 6.6 – 8.5), with 22 knees (53.7%) falling within the recommended range [16] (*p* < 0.001) (Fig. 3).

Lastly, in terms of axial alignment, Oxford UKA had a mean femoral rotation of 4.8° [standard deviation (SD) 3.4, range 0.3–15.4] external rotation in relation to the TEA. Five patients (6.2%) had external rotation of the femoral component of more than 10°. Fixed bearing UKA had a mean femoral rotation of 7.2° (SD 4.6, range 0.2–16.5), with 11 patients (28.2%) having an external rotation of more than 10°. Femorotibial rotational mismatch of more than 10° was noted in 13.8% in Oxford UKA and 20.5% in fixed bearing UKA (*p* = 0.04).

## Discussion

The results from our study show that Microplasty® instrumentation of Oxford UKA was more accurate compared to conventional fixed bearing UKA. One may argue that higher accuracy was seen with Oxford UKA as the reference range for the component position was wider. While this may be true, an implant design that is forgiving in tolerating a broader range of alignment is an advantage over designs that require strict component positioning. The spherical femoral component and spherically concave bearing in Oxford UKA has a much larger contact area compared with the fixed bearing design [11], so suboptimal alignment will less likely result in high contact stress and rapid wear [17]. This was evident in Gulati et al.’s series where they found no compromise in clinical outcome within 10° of malalignment in any direction of the femoral component [18]. In the sagittal plane, the reference range for the tibial component in Oxford UKA is also much wider than fixed bearing UKA. In contrast to fixed bearing UKA, where a tibial slope outside the range of 8 to 11° is associated with a worse outcome, it has been shown that a range of ± 5° of tibial slope has no effect of the clinical outcome in Oxford UKA [18]. With Oxford UKA, the initial tibial cut defines the tibial slope and the flexion gap. The extension gap is then chased by progressively removing the distal femur with the spherical mill. Therefore, with this gap technique, whatever the tibial slope, the flexion-extension gaps will be equal. Compared with the conventional fixed bearing instrumentation, where the components are applied anatomically, the flexion and extension gap will be affected by the tibial slope.

Moreover, our study also showed that the Oxford Microplasty® instrumentation enables greater consistency in the placement of femoral and tibial components when compared with conventional fixed bearing instrumentation. The use of an IM link in the Oxford Microplasty® instrumentation allows more reproducible placement of the femoral drill guide. For tibial resection, the coupling of the femoral gap-sizing spoon, the G clamp and the tibial saw guide places the femoral and tibial components in a more contiguous position, thus limiting the procedural approximations and consequent positional outliers [19].

There are several limitations to this study. Firstly, this series is limited to an Asian population; therefore, the findings may not be extrapolated to other ethnicities. Secondly, this study only evaluates the radiological outcomes (i.e., component positions), and further studies are needed to illustrate whether improved component positions correlate with better long-term implant survivorship. Thirdly, there were no bearing dislocations in this series, so no conclusion can be drawn regarding the correlation between improved instrumentation accuracy in reducing risk of bearing dislocation. Despite these limitations, this is the first cohort study comparing the radiographic data between Oxford mobile bearing Microplasty® instrumentation and conventional fixed bearing UKA instrumentation systems.

## Conclusion

In summary, we demonstrated that Microplasty® instrumentation for Oxford mobile bearing UKA was more accurate and precise compared to fixed bearing UKA with conventional instrumentation, in sagittal, coronal and axial alignment. Prospective studies with long-term follow-up are warranted to investigate the clinical implications.

## Data Availability

The datasets used or analyzed during the current study are available from the corresponding author on reasonable request

## Abbreviations

UKA: Unicompartmental knee arthroplasty
TKA: Total knee arthroplasty
AMOA: Anteromedial osteoarthritis
ACL: Anterior cruciate ligament
CT: Computed tomography
AP: Anteroposterior
TEA: Transepicondylar axis
KRA: Knee rotation angle
ICC: Intraclass correlation coefficient
SD: Standard deviation

## References

[1] Argenson JA, Chevrol-Benkeddache Y, Aubaniac J (2002). Modern unicompartmental knee arthroplasty with cement: a three to ten-year follow-up study. J Bone Joint Surg Am..

[2] Deshmukh RV, Scott RD (2001). Unicompartmental knee arthroplasty: long-term results. Clin Orthop Relat Res..

[3] Squire MW, Callaghan JJ, Goetz DD, Sullivan PM, Johnston RC (1999). Unicompartmental knee replacement. A minimum 15 year followup study. Clin Orthop Relat Res..

[4] Jenny JY, Boeri C (2003). Unicompartmental knee prosthesis implantation with a non-image-based navigation system: rationale, technique, case-control comparative study with a conventional instrumented implantation. Knee Surg Sports Traumatol Arthrosc.

[5] Muller PE, Pellengahr C, Witt M, Kircher J, Refior HJ, Jansson V (2004). Influence of minimally invasive surgery on implant positioning and the functional outcome for medial unicompartmental knee arthroplasty. J Arthroplasty.

[6] Weber P, Schroder C, Utzschneider S, Schmidutz F, Jansson V, Muller PE (2012). Does increased tibial slope reduce the wear rate of unicompartmental knee prostheses? An in vitro investigation. Orthopade.

[7] Gulati A, Weston-Simons S, Evans D, Jenkins C, Gray H, Dodd CA, Pandit H, Murray DW (2014). Radiographic evaluation of factors affecting bearing dislocation in the domed lateral Oxford Unicompartmental knee replacement. Knee.

[8] Kim SJ, Postigo R, Koo S, Kim JH (2014). Causes of revision following Oxford phase unicompartmental knee arthroplasty. Knee Surg Sports Traumatol Arthrosc.

[9] Lee SY, Bae JH, Kim JG, Jang KM, Shon WY, Kim KW, Lim KC. The influence of surgical factors on dislocation of the meniscal bearing after Oxford medial unicompartmental knee replacement: a case-control study. Bone Joint J 2014;96–b:914–22. 10.1302/0301-620X.96B7.33352 24986945

[10] Chatellard R, Sauleau V, Colmar M, Robert H, Raynaud G, Brilhault J (2013). Medial unicompartmental knee arthroplasty: does tibial component position influence clinical outcomes and arthroplasty survival?. Orthop Traumatol Surg Res.

[11] Goodfellow J, O’Connor J, Pandit H, Dodd C, Murray DW (2016). Unicompartmental arthroplasty with the oxford knee.

[12] Pandit H, Jenkins C, Gill HS, Barker K, Dodd CA, Murray DW (2011). Minimally invasive Oxford phase 3 unicompartmental knee replacement: results of 1000 cases. J Bone Joint Surg Br.

[13] Mohammad HR, Strickland L, Hamilton TW, Murray DW (2018). Long-term outcomes of over 8,000 medial Oxford Phase 3 Unicompartmental Knees - a systematic review. Acta Orthop..

[14] Peersman G, Stuyts B, Vandenlangenbergh T, Cartier P, Fennema P (2015). Fixedversus mobile-bearing UKA: a systematic review and meta-analysis. Knee Surg Sports Traumatol Arthrosc.

[15] Watanabe S, Sato T, Omori G, Koga Y, Endo N (2014). Change in tibiofemoral rotational alignment during total knee arthroplasty. J Orthop Sci.

[16] Hernigou P, Deschamps G (2004). Posterior slope of the tibial implant and the outcome of unicompartmental knee arthroplasty. J Bone Joint Surg Am.

[17] Psychoyios V, Crawford RW, O’Connor JJ, Murray DW (1998). Wear of congruent meniscal bearings in unicompartmental knee arthroplasty: a retrieval study of 16 specimens. J Bone Joint Surg Br.

[18] Gulati A, Chau R, Simpson DJ, Dodd CA, Gill HS, Murray DW (2009). Influence of component alignment on outcome for unicompartmental knee replacement. Knee.

[19] Koh IJ, Kim JH, Jang SW, Kim MS, Kim C, In Y (2016). Are the Oxford medial unicompartmental knee arthroplasty new instruments reducing the bearing dislocation risk while improving components relationships? A case control study. Orthop Traumatol Surg Res..

